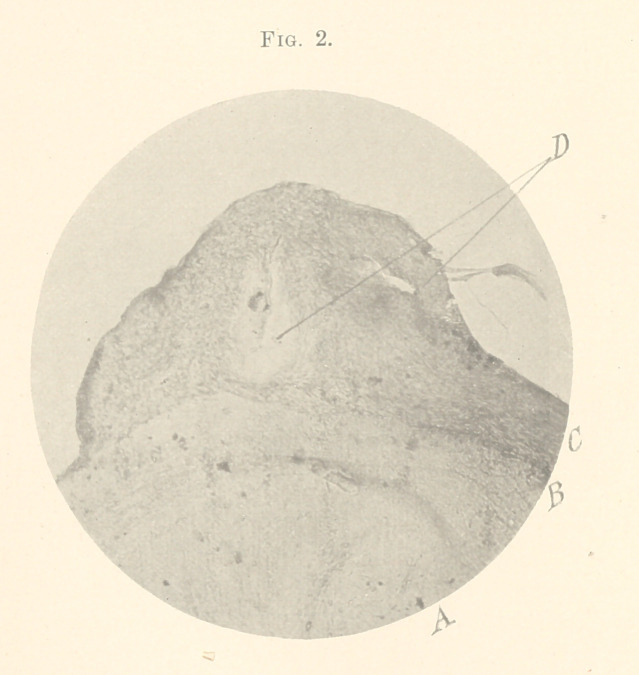# Pyorrhœa Alveolaris

**Published:** 1896-04

**Authors:** Eugene S. Talbot


### PYORRHŒA ALVEOLARIS.[1]

BY EUGENE S. TALBOT.[2]

Much literature of the present disease is chiefly given to its name, with the intent of fixing the views of the particular name-deviser on dental science.

The old up-hill scientific blunder of replacing names with meaning fixed by usage by others better coined from a linguist's standpoint leads only to the accumulation of names.

Riggs's disease is a title too firmly fixed to be dislodged by terms limiting the pathology of the disease, but expressive merely of temporary phases of dental thought.

Theories as to the etiology, pathology, and treatment of Riggs's disease are too numerous, discordant, and fleeting to merit lengthy discussion except as they seemingly express dominant thought. The latest theory advanced is the influence of the dyscrasic or constitutional abnormal states so much discussed in medicine, which has found expression in dental pathology in the uric-acid or gouty theory advanced by Dr. Reeves, and amplified by Dr. Peirce,[3] of Philadelphia. This has found support by able practitioners.

Clinical tests alone should be applied to all theories. Applying these to the uric-acid or gouty theory, I found that in but few cases of the uric-acid diathesis could any evidence suggesting pyorrhœa be detected.

According to Peirce, when uric-acid salts attain a certain percentage they are eliminated from the blood through the walls of the capillary vessels, passing out associated with lymph. The tissues become the seat of this salt exudate, and permeate

---

[1] Read before the Academy of Stomatology, January 28, 1896.

[2] Fellow of the Chicago Academy of Medicine.

[3] International Dental Journal, vol. xv. pp. 1, 217, 501.

the connective tissue, this tissue presenting the greatest density and least degree of vascularity. To test the validity of this claim of Dr. Peirce I had instituted two independent series of examinations: one at the Columbus Medical Laboratory, where special cases were examined; the other at the laboratory of the Northwestern University Woman's Medical School, to which teeth were sent as they were removed from the mouth. One hundred examinations were made in one, and one hundred and fifteen in the other. Teeth were obtained from three institutions in Chicago which make a speciality of extraction. I was careful in selecting only diseased teeth with calcic deposits. Of the one hundred examinations made in the Columbus Medical Laboratory fifty were specimens of calcic deposits from my patients; fifty were obtained at the institutions just mentioned, and therefore have no history.

The one hundred examinations at the laboratory of the Northwestern University Woman's Medical School have no history, except that they were good cases of pyorrhœa, with plenty of calcic deposits, loose in the sockets when extracted.

The three tests recommended by Dr. Peirce were used in both laboratories,—

1. The hydrochloric acid test.
2. The dry distillation test.
3. The murexid test.

Of the one hundred and fifteen examinations made at the Northwestern University Woman's Medical School by the first test, in only two cases was found the needle-shaped crystals, and one in which there was a slight resemblance of uric-acid crystals.

By the dry distillation test, thirteen gave no reaction from ammonia, and in seven the reaction was slight. The remaining eighty gave a decided reaction.

By the murexid test, four gave a slight murexid color, but the remainder gave no reaction.

Special examination was made of twelve of these teeth by the addition of strong hydrochloric acid, warming, decanting the acid, and washing with water. These gave no reaction by the dry distillation tests for ammonia. Two gave a slight reaction by the murexid test.

In examination of three uric-acid diathetic women, over forty years of age, uric acid was not detectable either by the murexid test or microscopically.

The examinations made in the Columbus Medical Laboratory are still more interesting, since among them are specimens from patients whose history could be obtained, and the results easily noticed.

Of the fifty obtained outside, eight gave positive results from all three tests. The other forty-two were positive by dry distillation, and negative by the murexid and microscopical tests. Of the fifty patients, thirty-eight females and twelve males, thirty-two are over forty years of age, twelve over thirty years, and six over fifteen years.

Twenty-six have uric acid to a greater or less extent.

Nine suffer with indigestion, seven of which are subject to sick headache.

Thirty-four have rheumatism.

Six are English, and four of these have the true English gout; the other two have rheumatism. All are positive with the dry distillation test. All are negative with the murexid test. Forty-nine are negative with the microscopical test. One shows needle-shaped crystals, but not uric acid. It is a singular fact that the cases examined in both laboratories, and in which there was uric acid and gouty histories, gave negative results.

Examination of calcic deposits by the dry distillation test shows that out of two hundred and fifteen cases, all, with the exception of the twelve cases which have been treated to remove all of the nitrogenous material, responded. The twelve cases so treated did not respond, since all nitrogenous compounds in and about teeth (even the saliva), when burned to an ash, will produce ammonia. This test is of no value. By the murexid test only twelve out of the two hundred and fifteen gave a positive reaction. By the microscopic examination only ten showed crystals. One of the chemists who made the examination is positive that they are uric acid crystals; the other is not, since lime-phosphate crystals resemble them too minutely to be with difficulty distinguished. Admitting that all of those cases reported be uric acid, it is barely possible that it might come from the blood direct, since blood-stains are almost always found upon the deposit, either from extraction or in removing it from the teeth in the mouth. Even if all the cases reported from both laboratories be uric acid deposits, this is a small percentage (about five per cent.) of cases in so large a number, and far from supporting the broad claim that the uric acid is the chief cause. The uric acid deposits are probably nothing more than an expression of the condition of the blood, and not the cause of the disease.

If this theory, moreover, were correct, the deposits, which are in the capillaries in a liquid state, surrounding the teeth, would attack all the teeth at the same time, and deposit equally or nearly so around the root of each tooth, which never occurs.

Certainly uric acid cannot produce that peculiar form of pyorrhœa in which no deposits are found, and which is so frequently observed. Again, there are many who suffer from uric acid poisoning for years, yet do not have tartar or serumal deposits. I have had uric acid poisoning for ten years, but have an exceptionally healthy mouth.

The uric acid poison theory does not account for those large, dense deposits frequently observed around the margins of cavities, protruding fillings, or points of irritation, and in no other locality in the tooth. The uric acid or gouty diathesis theory is not in harmony, moreover, with clinical observation, as I shall show later on.

Since pyorrhœa is so prevalent among our patients, and since so few suffer with uric acid poisoning, causes other than a gouty diathesis must be looked for.

Below are the official reports.

LABORATORY OF THE NORTHWESTERN UNIVERSITY.
WOMAN'S MEDICAL SCHOOL.

JEROME H. SALISBURY, A.M., M.D.,
Professor of Chemistry.

CHICAGO, December 14, 1895.

DR. EUGENE S. TALBOT:

DEAR DOCTOR,—I have examined the calcic deposit from one hundred teeth by the three tests which you suggested,—viz., dry distillation, the murexid test, and microscopic examination of the residue after evaporation with hydrochloric acid.

By the dry distillation test thirteen gave no reaction for ammonia, and in seven the reaction was slight. The remaining eighty gave a decided reaction.

By the murexid test four gave a slight murexid color, but the remainder gave no reaction.

Under the microscope I saw in two cases needle-shaped crystals, and in one some crystals which somewhat resembled the crystals of free uric acid. In the other specimens I found no indication of uric acid.

I have examined twelve other teeth by the same tests, except that I preceded the dry distillation by the addition of strong hydrochloric acid, warming, decanting the acid, and washing with water. These gave by dry distillation no reaction for ammonia. By the murexid test two gave a slight reaction.

The specimens of calcic deposits from the cases of Mrs. T., Mrs. C., and Mrs. H., gave no indication of uric acid by either the murexid reaction or microscopic examination.

As a test for uric acid the method of dry distillation, and testing the gases evolved for ammonia, is in my opinion entirely inconclusive. The reaction will of course be given by most nitrogenous organic matters, and some of these will always be present in the material taken from the teeth.

The murexid test is not entirely conclusive, as there are a few other substances that give a similar reaction, but they are not very likely to be present in the calcic deposit on the teeth. The murexid reaction obtained in the tests which I made may have been given by the dried blood, as blood sometimes contains enough uric acid to give a decided reaction.

The discovery of needle-shaped crystals by the microscope cannot be regarded as positive evidence of the presence of calcium urate, because calcium phosphate crystallizes in crystals of similar shape.

Yours very respectfully,
(Signed) J. H. Salisbury,
Professor of Chemistry,
Northwestern University, Woman's Medical School.

Columbus Medical Laboratory,
Columbus Memorial Building, Suite 1403,
103 State Street. Tel. Main, 3866.

A. Gehrmann, M.D., Bacteriologist.
John A. Wesener, Ph.C.H.D., Chemist.
W. Evans, M.D., Pathologist.
Wm. M. Harsha, M.D., Secretary.

Chicago, December 20, 1895.

Dr. E. S. Talbot, No. 1205, 103 State Street, Chicago:

Dear Doctor,—I beg leave to report as follows on the specimens of incrustations from one hundred teeth and calcic deposits sent the laboratory. The tests employed were,—

No. 1, dry distillate.
No. 2, murexid.
No. 3, crystals.

Each of these tests was employed in each of these cases. One hundred responded to test No. 1. Eight responded to tests Nos. 2 and 3.

Test No. 1 gives a reaction with any organic compounds giving ammonia as a decomposition product. Saliva ash gives the reaction.

The conclusion to be drawn, then, is that eight specimens of this one hundred contained uric acid and urates.

Very truly,
Dr. J. A. Wesener,
Chemist to Columbus Medical Laboratory.

Ten years ago[1] I published some observations on pyorrhœa, which it was hoped some one proficient in microscopy would take up, and pursue the investigations to the end. I have never observed any allusion to it. Among the points then made was that pyorrhœa was on the increase; that a large majority of patients suffered more or less from it, and that modern dentistry had most to do with the cause.

I still hold these opinions, since clinical experience in the different asylums of this country and Europe, as well as close observa-

[1] Dental Cosmos, 1886, vol. xxvi. p. 689.

tion of my office patients, has given me a broad range to study the etiology of this disease.

Dr. E. Magitot in 1867 published the most complete paper upon this subject, describing the disease in its progress to the end, but says that the gum, being in all cases attacked subsequently only, is not the real seat of the lesion. The disease with which we are occupied, he says, "seems essentially characterized, from an anatomical point of view, by a slow and progressive destruction of the periosteal membrane,—a destruction of an inflammatory character, of chronic progress, proceeding from the neck to the end of the root, and leading without fail to the loss of the tooth. This special feature, its mode of origin, and the precise seat of the lesions seem to justify the name alveolo-dental periostitis. But, notwithstanding its primary origin in the periosteum, and its complications with the gum and bony alveolar wall itself, the study of the successive morbid phenomena does not allow us to admit, as various authors have claimed, that these parts are originally the seat of the disease. I believe Dr. Peirce takes the same view. I take just the opposite,—

1. That the gum is the first tissue attacked, and,

2. That these parts are originally the seat of the disease.

When a tooth has been extracted that has been associated with pyorrhœa, clinical observation shows that the margin of the peridental membrane has changed its locality. Instead of being in its normal position at the neck of the tooth, it has receded in a more or less irregular line towards the apex of the root. The extent of this recession depends upon the duration and power of resistance of the disease.

The membrane, instead of being thin and of a pink color, is quite thick and of a deep red. The inflamed membrane may extend through the entire length of the root, or in circumscribed localities. The space upon the root made vacant by the destruction and loss of the peridental membrane may be quite clear and smooth, or it may possess calcic deposits. These deposits may consist of a uniform ring extending around the entire tooth, or circumscribed masses of deposits may be located at different points about the root. As the membrane recedes, the deposits follow after it upon the root or roots.

Between the border of the calcic deposits and the peridental membrane is a space one to two lines in width, where the root or roots of the tooth is perfectly smooth; this is the pus-line of clear, smooth tooth-structure, and is nearly always situated between calcic deposits and the peridental membrane, showing that the calcic deposits are not always found in the membrane as Dr. Peirce claims. 2. The space upon the root of the tooth between the peridental membrane and the neck of the tooth, including the calcic deposits, is bathed in pus. This disease is an inflammation of the gums, and is due to irritation from constitutional and local causes.

Constitutional causes are tartar, from mercurial salivation, potassium iodide, and other drugs, syphilis, loss of vitality, locomotor ataxia, paretic dementia, and the menstrual nisus. In neurotic and degenerate classes, as a whole, pyorrhœa exists to a greater extent than in the more healthy classes. In any and all of those diseases in which systemic disturbances produce trophic changes this disease is present.

### LOCAL CAUSES.

As has already been said, modern dentistry is producing more pyorrhœa than any other one cause. Some cases result from infection, from micro-organism, application of the rubber-dam, clamps, wedging the teeth, correcting irregularities, sharp edges of decayed or filled teeth, protruded fillings, spaces between teeth, crown- and bridge-work, over-stimulation in the use of the toothpick, artificial teeth, more particularly ill-fitting plates, injuries, tartar-accumulation and decomposition of food and collections around the necks of teeth, tobacco, and everything of a foreign nature, as observed in the mouths of idiots, imbeciles, epileptics, and all individuals who do not take care of the teeth. The result of irritation from constitutional and local causes is inflammation.

Light is thrown upon this subject by a careful study of the anatomy and physiology of the parts involved. We have the root or roots of a tooth on the one hand and the bony structure of the alveolar process on the other, and between the two resisting walls we have the peridental membrane, composed of fibrous elastic connective tissue, which give nourishment to both the tooth and the alveolar process. The alveolar process is a transient bony structure, simply for the purpose of holding the teeth in place after they have erupted. The gums or mucous membrane which covers the alveolar process, and which is united with the mucous membrane throughout the mouth, connects with the peridental membrane at the margin of the process. That the lymphatic system is richly developed at this locality is demonstrated by the fact that when the temporary and permanent teeth are lost the alveolar process absorbs. No structure of the body is similarly situated as the peridental membrane. The structure of the tooth, not changing its form or size, sends very little nourishment into the cementum.

The peridental membrane obtains its blood-supply from the arteries at the apex of each root, just before they enter the foramen and through the alveolar process, but the largest amount passes through the gingival border of the gum.

According to Black[1] these capillaries run longitudinally from either end of the root towards the centre, giving off branches which enter the alveolar process, but not the cementum, because of the peculiar locality of the membrane. May not the anatomical position and a physiological action on this membrane have something to do with the disease?

The gums are rarely found in a healthy condition. They may become inflamed from either constitutional or local causes mentioned above. If the cause is removed early and antiseptic and astringent washes used, together with the stimulating effect of the tooth-brush, the gums will return to a healthy condition. The peridental membrane is never invaded by pus-germs so long as it is in a perfectly normal state. If, on the other hand, inflammation of the gums, due to either constitutional or local causes, persists, it will extend to the capillaries of the peridental membrane, causing inflammation and stasis of blood in that direction.

The peridental membrane has not lost all of its source of nourishment, although the greater part is cut off; its vitality is thus impaired.

When inflammation of the peridental membrane takes place, a proliferation of small round cells produce a new connective tissue. This tissue causes inflammation and thickening of the peridental membrane, which is subject to necrosis, first, from its position between the two bony walls, causing pressure; and secondly, from deficient blood-supply.

Atheromatous patches, composed of granular *débris* and fatty detritus, in which are deposited lime-salts liberated from the tissue-cells and from the blood or lymph, are then formed. These patches soon become infected with pus-germs, or infection of the tissue in the primary stage of the inflammation may take place.

These pus-germs, according to Miller, are found in every mouth, but more especially around the necks of the teeth. Infection means degeneration and liquefaction, not only of the immediate tissue,

---

[1] American System of Dentistry.

but also of the more healthy peridental membrane, but in a less marked degree.

Pus-infection-producing pockets are formed first by circumscribed inflammation at a particular point of the gum or peridental membrane at the neck of the tooth. The inflammatory process extends into the peridental membrane along a blood-vessel or lymph-stream. This may extend part of or the entire length of the root of the tooth, the tissue-degeneration taking place in precisely the same manner as before, only in a circumscribed way. In phthisical patients, and those with low vitality, and patients who have been ill for any length of time, a low form of inflammation of the gums extending to the peridental membrane with pus-infection takes place, and degeneration of tissue ensues, with or without granular patches and calcic deposits.

The granular *débris* or calcic deposits in all cases are a secondary consideration in this breaking down of tissue, the inflammatory exudate and pus-formation being primary.

Sometimes the degeneration of tissue will extend the entire length of the root. The atheromatous patch of degeneration is always located in that part of tissue farthest from the blood-supply or at the point of least vitality, hence the reason of the breaking down of membrane and deposit upon the root of the tooth.

A pathological condition of the jaw, familiar to all dentists, which must here be described, although seemingly foreign to the subject, is due to the immediate cause of pyorrhœa. Its etiology has never been explained. I refer to that condition of the jaw when the pulp is dead in the tooth and a large or small area of the bone about the root has absorbed; frequently the bone is entirely lost about the root and the cavity covered over by the external mucous membrane. Upon opening this cavity the root or roots are exposed to a greater or less extent, depending entirely upon the size of the cavity.

When death of the pulp takes place, with or without alveolar abscess, inflammation of the peridental membrane may take place at the apex of the root or roots. We now have the same process as before the pus-infection and degeneration of tissue, the pus-germs infecting the peridental membrane through the pulp-canal. Absorption and liquefaction of the alveolar process takes place after the death of the peridental membrane (not due to pressure of an alveolar abscess, but from pus and natural absorption due to the destruction of the peridental membrane), and the root is frequently partly or wholly covered with calcic deposits.

Lime-salts are chemically non-irritating, as cementum is properly formed from the peridental membrane, and as exostoses are found on the roots of teeth without having caused any symptoms, calcic salts deposited here are physiologically inert as any foreign material can be in the body. This is to be expected since lime-salts in the blood is nature's great weapon with which to ward off and heal disease and not to disorganize tissue.

That many forms of micro-organisms are present in the mouth, producing the original inflammation of the gums, there can be no doubt, since the cultures are there with favorable surroundings. They assist in producing the inflammatory process for the following reason: treat a mouth with one, two, or three loose teeth, heal the tissues, and allow the loose teeth to remain. Stop the treatment, except the free use of the brush, and in the course of a few weeks the inflammation can be seen starting from the loose teeth and extending from one tooth to another until all have become infected. Remove the tooth or teeth, and when the gums are in a healthy condition they will remain so by the same line of treatment. I have observed this many times.

The neurotics and degenerates, whether wealthy people or confined in State institutions, are mostly afflicted.

The age at which this disease begins favors those over forty years, but it is found in children. I have observed it in my little patients before having their teeth regulated. This is due, first, to trophic changes, and, secondly, to inflammation of the gums due to want of hygienic measures. I have also observed it in the mouths of asylum children, due to the same cause. Miller mentions " many rachitic children, from four to six years, who had but a few teeth left, and, also, " out of twenty-six cases under twelve years, in which seven manifested pronounced symptoms of pyorrhœa," the class of food given asylum patients of all ages, together with a want of cleanliness, produces the inflammation of the gums, following with pyorrhœal symptoms. A change in the management recently in a public institution near Chicago, and the appointment of an economical superintendent, caused the inmates to come down with scurvy, from the result of which a large number of cases of pyorrhœa developed. Patients suffering with locomotor ataxia and paretic dementia are very prone to this disease. This is true to trophic changes. In forty-four locomotor ataxics, all had the disease in a more or less marked degree. Of three hundred and sixty-five paretic dements, fully two-thirds had pyorrhœa.

A marked illustration of pyorrhœa due to trophic changes and want of hygienic measures is found in the mouths of pregnant women, and a most marked illustration due to the same cause is in domestic animals or wild ones in captivity.

Some authors have tried to associate pyorrhœa with catarrh and nasal lesions, such as polypi, adenoid vegetation, and hypertrophy of the mucous membrane, turbinates, and stenosis of the nasal cavity, as well as tonsillitis and all forms of sore throat. In a general way these lesions are found among the degenerate classes, and while one is not dependent upon the other, they are frequently associated, often patients suffering with pyorrhœa who claim to have been salivated. It can now be seen how such conditions can be brought about.

The period of life between forty and sixty years is to man a period of involution, when certain functions are ceasing to be active factors in the light of the individual. The structures devoted to retrograde metamorphosis assume predominance, and the arterial system shows a tendency to fatty changes. In both sexes, at this period, as at puberty, there is a marked tendency to nervous diseases. The changes of what well may be called the climacteric are the results of deficient power to supply nutrition rather than excess in utilization. Hence the primary change of the arteries and their secondary consequence in the peridental membrane. The gums become inflamed, and there is no desire to keep the mouth in a hygienic condition, hence pyorrhœa is more frequent in advancing years.

As the peridental membrane recedes the teeth loosen. The irritation in mastication assists in maintaining the inflammation, and the destruction of the membrane is hastened until the tooth becomes a foreign body and is exfoliated.

The alveolar process, as has been shown, is a transitory structure, simply for the purpose of holding the teeth in place; when they are removed the process absorbs. It is reasonable to suppose that when the peridental membrane is destroyed the process has lost its function and naturally absorbs away. This is hastened by the accumulation of pus in and about the process.

In a general way pyorrhœa, like all diseases of the body, is influenced to mildness or severity according to health and tonicity.

TREATMENT.

No one in the profession is more disappointed as to the views as to what shall constitute the line of treatment than the writer, since he has always advocated that pyorrhœa was a constitutional disease and required similar treatment. Investigation has shown that inflamed gums are due to constitutional and local causes. It is another illustration of the fact, however, that to know the cause is to find a remedy. It does not necessarily follow that the cure is an easy one to accomplish, since the location of the disease is nearly always difficult to reach. Especially is this so in the later stages. The line of treatment in the early stages is clear. It must be purely of a prophylactic nature.

No matter of what nature the patient may be; a monkey in the zoological gardens, a pet lap-dog, wild animals in captivity, rachitic and idiotic children in public institutions, or a degenerate in one of our best families, living in luxury without fresh air and outdoor exercise, and upon food entirely at variance with what they require, must necessarily have soft spongy gums. If the food be changed in animals, with a vigorous use of the tooth-brush, together with antiseptic and astringent washes, with plenty of exercise by our patients, the gums will remain in a healthy condition, the peridental membrane will not become involved, and pus-germs will not invade the tissue. In other words, healthy gums will invariably prevent the disease.

If inflammation has extended to the peridental membrane, and pus-infection has taken place, with or without calcic deposits, instruments should not be used so as to come in contact with the edge of the membrane. The membrane should not be injured under any circumstances. The slightest injury increases the area of the infection. Since inflammation and pus-infection are the primary stages of the disease, and the calcic deposits always secondary and a result, and since nature tolerates lime deposits in all parts of the body, it would seem immaterial whether the deposits are removed from the root or not, provided the application extended beyond the deposits and reached all parts of the diseased membrane. I know of no instance in medicine or surgery where the calcic deposits in any other part of the body are taken into consideration in the treatment of disease. Nature takes care of such deposits. Such being the case, there is no reason why nature will not tolerate calcic deposits on the root of a tooth. I do not, at this time, make the positive assertion that it is unnecessary to remove the deposits, since I have not had sufficient experience to warrant this statement, but simply offer it as a suggestion. From our past experience in the treatment of the disease, the deposits must be removed; and right here I would suggest that in the future treatment of this disease a dissolving fluid that is not injurious to the surrounding

Fig. 1.

Fig. 2.

tissue should take the place of instruments, especially when the disease is extensive.

What is necessary, whether we remove all the deposit or not, is to use an antiseptic disinfectant and germicide in such quantities that all parts of the tissue, including the deposit from the gingival margin of the gum to the edge of the peridental membrane, shall become perfectly immune. If this can be carried beyond the calcic deposits so as to reach the edge of the membrane and restore it to a healthy condition, we have accomplished all that is necessary, since the cause of the deposit will be removed.

It is readily understood how, if the disease is allowed to progress, the farther the edge of the membrane is from the gum-margin the more difficult it is to get the drug to the edge of the membrane, hence the necessity of early treatment. The farther the disease extends, the less the blood-supply to the membrane and the less show for recuperation. I cannot recommend any particular antiseptic, disinfectant, or germicide in the successful treatment of this disease. I have had marked success by saturating the gum thoroughly inside and out with officinal tincture of iodine every other day.

The following illustrations imperfectly show the changes which take place in the peridental membrane.

Figs. 1. and 2. show the root of a cuspid tooth: *A*, the dentine; *B*, the cementum; *C*, the peridental membrane.

The peridental membrane in Fig. 1. shows the first stages of inflammation, the infiltration of the round cells, and thickening. Fig. 2. shows the thickened peridental membrane, and the atheromatous degeneration marked *D*.

## Figures and Tables

**Fig. 1. f1:**
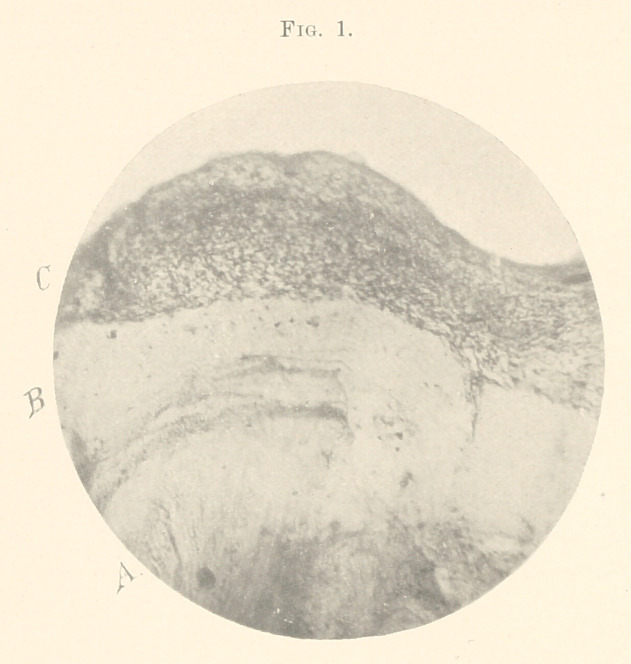


**Fig. 2. f2:**